# Population of American Cities

**Published:** 1871-01

**Authors:** 


### POPULATION OF AMERICAN CITIES.

| | 1870. | 1860. |
|---|---|---|
| New York | 927,436 | 813,659 |
| Philadelphia | 657,179 | 562,549 |
| Brooklyn | 406,097 | 266,714 |
| St. Louis | 312,963 | 150,780 |
| Chicago | 299,370 | 109,260 |
| Baltimore | 267,599 | 212,418 |
| Boston | 253,984 | 177,812 |
| Cincinnati | 218,900 | 161,041 |
| New Orleans | 184,688 | 173,780 |
| San Francisco | 158,361 | 56,801 |
| Buffalo | 114,247 | 81,123 |
| Washington | 109,338 | 611,13 |
| Cleveland | 93,018 | 43,411 |
| Pittsburgh | 86,255 | 47,218 |
| Jersey City | 82,630 | 43,884 |
| Detroit | 79,619 | 43,417 |
| Milwaukee | 71,464 | 45,246 |
| Albany | 69,452 | 62,367 |
| Providence, R. I. | 68,870 | 50,366 |
| Rochester, N. Y. | 62,424 | 50,938 |
| Alleghany City | 53,185 | 29,702 |
| New Haven, Conn. | 50,886 | 39,268 |
| Charleston, S. C. | 48,431 | 51,240 |

| | 1870. | 1860. |
|---|---|---|
| Troy, N. Y. | 46,471 | 39,235 |
| Syracuse, N. Y. | 43,061 | 26,342 |
| Worcester, Mass | 41,168 | 42,960 |
| Lowell, Mass | 40,937 | 39,027 |
| Indianapolis | 40,936 | 18,611 |
| Cambridge, Mass | 39,650 | 26,060 |
| Scranton, Pa | 38,762 | 9,223 |
| Hartford, Conn | 37,835 | 29,152 |
| Reading, Pa | 34,004 | 23,062 |
| Kansas City | 32,296 | .... |
| Toledo, Ohio | 31,693 | 13,758 |
| Columbus, Ohio | 31,336 | 18,554 |
| Wilmington, Del | 30,904 | 21,258 |
| Dayton | 30,866 | 20,081 |
| Lawrence, Mass | 29,992 | 17,630 |
| Charlestown, Mass | 28,330 | 25,063 |
| Lynn, Mass | 28,231 | 19,083 |
| Fall River, Mass | 26,768 | 14,016 |
| Springfield, Mass | 26,706 | 15,199 |
| Quincey, Ill | 24,363 | 13,032 |
| Salem, Mass | 24,119 | 22,252 |
| Manchester, N. H | 23,509 | 20,107 |
| Peoria, Ill | 22,864 | 14,045 |
| Evansville, Ind | 21,830 | 11,484 |
| New Bedford, Mass | 21,232 | 22,300 |
| Oswego, N. Y. | 20,960 | 19,288 |
| Leavenworth, Kan | 20,665 | 7,429 |
| Lancaster, Pa | 20,161 | 17,603 |
| Davenport, Iowa | 20,147 | 11,267 |
| St. Paul, Minn | 20,645 | 10,401 |

| | | | |
|---|---|---|---|
| London | 3,214,000 | Glasgow | 401,000 |
| Paris | 1,950,000 | Madrid | 390,000 |
| Constantinople | 1,500,000 | Dublin | 362,000 |
| Berlin | 800,000 | Manchester | 350,000 |
| St. Petersburg | 667,000 | Lisbon | 340,000 |
| Naples | 600,000 | Amsterdam | 250,000 |
| Vienna | 640,000 | Rome | 215,000 |
| Liverpool | 520,000 | Jerusalem | 20,000 |
| Moscow | 420,000 | Athens | 50,000 |

